# Occurrence of Internal Parasites and Anthelmintic Resistance in Goats

**DOI:** 10.3390/ani15071024

**Published:** 2025-04-02

**Authors:** Gianluca D’Amico, Adrian-Valentin Potârniche, Bianca-Irina Tucă, Adriana Györke

**Affiliations:** 1 Department of Parasitology and Parasitic Diseases, Faculty of Veterinary Medicine, University of Agricultural Sciences and Veterinary Medicine of Cluj-Napoca, 400372 Cluj-Napoca, Romania; gianluca.damico@usamvcluj.ro (G.D.); adriana.gyorke@usamvcluj.ro (A.G.); 2 Department of Infectious Diseases, Faculty of Veterinary Medicine, University of Agricultural Sciences and Veterinary Medicine of Cluj-Napoca, 400372 Cluj-Napoca, Romania

**Keywords:** goats, internal parasites, strongyles, anthelmintics, resistance, polymerase chain reaction (PCR)

## Abstract

Extensive anthelmintic (AH) use for endoparasitism prevention, especially gastrointestinal strongyle (GIS) infections, led to AH resistance (AR). This study assessed the internal parasitic profile and AR occurrence in goat herds. Five herds (H, n = 300) raised in household systems in Romania (2020–2021) were dewormed with eprinomectin in the fall (H1, H2) and albendazole in the spring (H3–H5). Fecal samples (n = 67) were collected pre- and 14 days post-AH administration. Coprological examinations, coprocultures, and polymerase chain reaction (PCR) were performed. Anthelmintic resistance was detected in H1, H2, and H5. Pre-AH administrations, *Eimeria* spp. was the most common parasite (95.5%), followed by GIS (91.0%), *P. rufescens*/*M. capillaris* (77.6%), *Dyctiocaulus filaria* (56.7%), and *Moniezia* spp. (43.3%). *Teladorsagia circumcincta* (4/5), *Trichostrongylus colubriformis* (4/5), *Oesophagostomum venulosum* (3/5), *Haemonchus placei* (3/5), *Haemonchus contortus* (2/5), and *Cooperia oncophora* (2/5) were identified by PCR. Mean OPG/EPG values for *Eimeria* spp. and GIS were <1000 eggs/gram feces, tending to be higher in young versus adult goats and in autumn versus spring. FECR (90%CI) values post-AH administration were 38.42 (23.4; 71.8), 54.71 (−4.1; 75.1) for eprinomectin, and 9.78 (−1.7; 64.8) for albendazole, suggesting AH ineffectiveness and AR presence in H1, H2, and H5, respectively. These findings highlight that specific management measures are needed.

## Simple Summary

The economic impact of endoparasites on goat production is a major concern, mainly because gastrointestinal strongyles (GIS) infections result in poor animal welfare, decreased productivity, and high veterinary costs. This has led to the large-scale administration of anthelmintics for prophylaxis but has also contributed to the development of resistance to anthelmintics. Spatial determination and reporting of endoparasite population and their degree of resistance are essential for effective monitoring and control, and farmers’ education. In the current study, among 300 goats originating from 5 herds in Romania, 95.5% were positive to *Eimeria* spp., 91.0% for GIS, and 77.6% for *P. rufescens*/*M. capillaris*, 56.7% for *Dyctiocaulus filaria*, and 43.3% for *Moniezia* spp., as assessed by coprological examination. *Teladorsagia circumcincta*, *Trichostrongylus colubriformis*, *Oesophagostomum venulosum*, *Haemonchus placei*, *Haemonchus contortus*, and *Cooperia oncophora* were also detected by polymerase chain reaction (PCR). Resistance to the anthelmintics eprinomectin and albendazole was detected, with variations across herds in terms of the anthelmintic level. These variations underline that herd-specific management measures are needed. Parasite control should be based on coproparasitological examination. Uncontrolled and excessive use or underdosing of anthelmintics should be avoided.

## 1. Introduction

Infections with internal parasites affect animal health, posing a major economic burden for farmers and farming industries, particularly in communities with limited resources [1,2]. In goats, the most common internal parasites are classified as gastrointestinal nematodes (roundworms), protozoan parasites, cestodes (tapeworms and their larval forms), trematodes (flukes), and lungworms [3]. Among these, gastrointestinal strongyles (GIS) represent a significant challenge as they cause clinical diseases and productivity loss [1,4]. The most common GIS affecting small ruminants are *Haemonchus*, *Trichostrongylus*, *Ostertagia*, *Cooperia*, *Bunostomum*, *Oesophagostomum*, *Chabertia*, and *Nematodirus* [5,6,7,8,9]. They can decrease feed digestibility and nutrient absorption, resulting in malnutrition, weight loss, and weakness, and produce chronic diarrhea, leading to dehydration and electrolyte imbalances. In addition, some nematodes, such as *Haemonchus contortus*, feed on the host’s blood, causing severe anemia [5,10].

The introduction of prophylactic anthelmintic (AH) treatment using benzimidazoles, macrocyclic lactones, and imidazothiazoles has achieved unprecedented levels of parasite control and significantly improved animal health and production [2,11]. AHs have proven effective with a good safety profile and a broad spectrum of activity, in addition to being affordable, leading to widespread adoption and use [2]. However, this has led to the development of resistance to AHs (AR) [12]. Even though resistance genes are initially rare in parasite populations, their frequency increases as selection progresses, increasing the number of resistant parasites [13]. Various published studies in small ruminants have documented AR among GIS, including multidrug resistance (MDR) [2]. GIS MDR was reported to fenbendazole, ivermectin, levamisole, and eprinomectin in Poland [11,14,15], to benzimidazoles and ivermectin in the Czech Republic [16], to albendazole and ivermectin [17], and to benzimidazoles, ivermectin, and levamisole in Slovakia [18], and to benzimidazole and eprinomectin in France [19,20]. In Romania, there is only one report of GIS resistance to macrocyclic lactones (eprinomectin) and benzimidazoles (albendazole) in a goat herd [21].

Since the administration of AHs remains the backbone of controlling GIS infections in small ruminants worldwide, AR seriously threatens these animals’ health, welfare, and production [11]. This poses a substantial challenge to the long-term sustainability of livestock farming. The spatial determination and reporting of AR are crucial for efficient parasite control, monitoring, research, policy-makers, and educational initiatives to manage and mitigate the impact of AR.

This study was designed to provide an overview of the occurrence of internal parasites and GIS species in goats raised in household systems in northwestern Romania and to evaluate the outcomes after routine prophylactic AH use against GIS by assessing AR.

## 2. Materials and Methods

### 2.1. Experimental Design

This study was conducted in five goat herds raised in extensive systems, mainly for milk production, located in the northwestern part of Romania (Someșan and Western Târnavelor Plateaus). These areas are part of the Transylvanian Plateau and have a hilly character, situated at altitudes of 500–600 m and 300–400 m, respectively. The climate in this area is continental, featuring four distinct seasons. During the study, outdoor temperatures ranged from roughly 0 °C to 15 °C. The fall season was characterized by declining temperatures and moderate precipitation, including occasional rain, while the spring season was characterized by rising temperatures and variable precipitation, such as sporadic rain and the chance of late snowfalls, particularly in March [22]. Animals grazed mostly on the same pastures between March and November for 8–10 h per day. In the colder months, they were housed in wooden stables and fed with hay and corn. The medical records from these herds show that all adult goats have been routinely dewormed twice a year at scheduled intervals. During the autumn, they were treated with ivermectin at a dose of 0.2 mg/kg administered via subcutaneous injection, while in the spring, albendazole was administered orally at a dose of 10 mg/kg.

**Figure 1:**
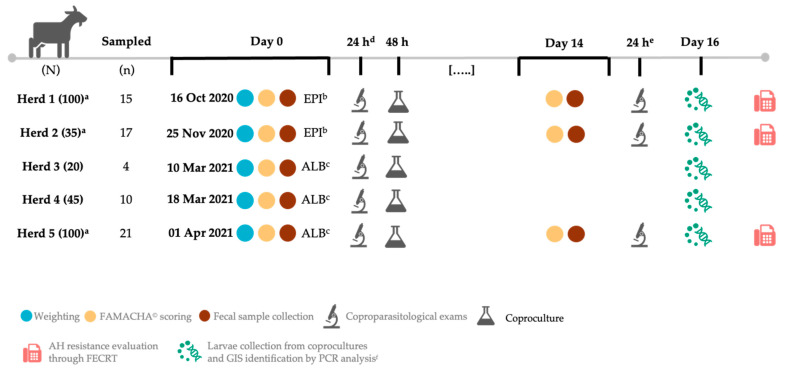
Study design. ^a^ Herds selected for assessment of AR occurrence based on Coles et al. (1992) [23] and Kaplan et al. (2023) guidelines [24]; ^b^ 1 mg/kg pour-on (Eprinex multi 5 mg/mL, Boehringer Ingelheim, France); ^c^ 10 mg/kg per os (Dufalben 10%, DutchFarm International, Netherlands); ^d^ flotation, sedimentation, and Baermann methods, followed by OPG/EPG count by the McMaster method (as applicable); ^e^ McMaster method only; ^f^ PCR was performed within 30 days after larvae collection. AH, anthelmintic; GIS, gastrointestinal strongyle; FECRT, fecal egg count reduction test; PCR, polymerase chain reaction.

At the time of study inclusion, the herds totaled 300 animals and were distributed as follows: H1 (n = 100), H2 (n = 35), H3 (n = 20), H4 (n = 45), and H5 (n = 100). Routine anthelmintic prophylaxis was performed in October-November 2020 (fall season) and March–April 2021 (spring season) as established by the local veterinarian. More details on herd size and sampling are included in Figure 1.

Of the total goats, 67 were randomly selected for coproparasitological evaluation, being distributed as follows to reach at least 15% of the herd size: H1 (n = 15), H2 (n = 17), H3 (n = 4), H4 (n = 10), and H5 (n = 21). The selection criteria included clinically healthy animals that were at least six months of age and had not received AHs in the eight weeks prior to the study initiation [23]. All herds were included in the analysis of internal parasite occurrence. Only herds H1, H2, and H5 met the requirements outlined by the World Association for the Advancement of Veterinary Parasitology (WAAVP) guidelines for AR identification [23,24]. Therefore, these herds were included in the AR analysis. H3 and H4 were excluded because the sample size and egg counts were not compliant with these guidelines.

The selected goats were identified by the ear tag, individually weighed, and examined for anemia. Anemia was assessed using the FAMACHA^©^ (Baton Rouge, LA, USA) scoring system, a tool used to make deworming decisions for haemonchosis in small ruminants based on different levels of anemia [25]. Then, approximately 10 g of fecal sample was collected from each goat, directly from the rectum before (day 0) and 14 days after AH administration. Each sample was labeled in a plastic bag for animal identification and stored at 4 °C until coproparasitological examination.

AHs were administered as follows: eprinomectin pour-on at 1 mg/kg dose (Eprinex multi 5 mg/mL, Boehringer Ingelheim, Paris, France) in herds H1 and H2, and albendazole per os at 10 mg/kg dose (Dufalben 10%, DutchFarm International, Nederhorst Den Berg, The Netherlands) in herds H3, H4, and H5 (Figure 1).

Sample processing included coproparasitological examination performed within 24 h after sample collection at both time points (days 0 and 14). To identify GIS species, fecal samples from positive animals were grouped by herd and cultured. The 3rd-stage larvae (L3) obtained were subjected to DNA extraction (a minimum of 10 larvae per herd), and PCR analysis was performed within 30 days to identify 13 GIS species.

### 2.2. Coproparasitological Examination

The fecal samples were analyzed using coproparasitological techniques such as flotation with saturated NaCl solution (density 1.18), sedimentation, and the Baermann method [26]. Parasitic elements (oocysts, eggs, and larvae) were identified based on their morphological characteristics under light microscopy (Novex®, Arnhem, The Netherlands) [26]. The number of *Eimeria* spp. oocysts (OPG) and gastrointestinal nematode eggs (EPG) per gram fecal sample were established by the McMaster method [26]. No *Moniezia* spp. eggs were counted, but their presence was recorded.

Further, five grams of feces from GIS-positive samples were pooled in one sample by herd. Coproculteres were performed using the method described by Reinecke [27] to obtain L3 of GIS. The larvae collection was performed using the technique used by Euzéby in 1982 [28]. Larvae were stored in a refrigerator at 4 °C until further examination by PCR for GIS species identification.

### 2.3. GIS Species Identification by PCR

DNA was extracted from 200 μL larvae suspension obtained by coprocultures using the commercial Isolate II Genomic DNA Kit (Bioline, London, UK), according to the manufacturer’s instructions. Amplification of the barcode region of ITS-2 of various GIS species was performed using the primers shown in Table 1. PCR reaction mixtures of 25 μL were prepared, containing 5 μL PCR mix (FIREPol^®^ Master Mix, Solis ByoDine, Tartu, Estonia), 15 μL of DNase/RN ase-free distilled water (Promega, Madison, WI, USA), 0.5 μL of each primer, and 4 μL of the DNA sample. Amplification was performed in a thermal cycler (Bio-Rad C1000™ Thermal Cycler, Bio-Rad Laboratories, Hercules, CA, USA). The reaction conditions were as follows: 94 °C for 8 min, followed by 35 cycles of 94 °C for 30 s, 50–62 °C for 30 s, and 72 °C for 1 min, and a final elongation step of 72 °C for 7 min. Amplification products were visualized by gel electrophoresis in 2% agarose gel stained with SYBR™ Safe DNA Gel Stain (Invitrogen by Thermo Fisher Scientific, Cambridge, UK). To visualize the presence of specific fragments, the gel was examined using the Bio-Rad BioDoc-It™ ImaGISg System (Bio-Rad Laboratories, Hercules, CA, USA). The DNA fragment length was compared with a 100 bp molecular marker (GeneRuler 100 bp DNA Ladder, Fermentas, Waltham, MA, USA) and interpreted according to Table 1. Ultra-pure distilled water was used as a negative control.

### 2.4. Evaluation of AR Occurrence

Occurrence of AR was evaluated using a fecal egg count reduction test (FECRT) as described in the WAAP guidelines. A paired study design for clinical studies was applied to herds H1, H2, and H5 [23,24]. Fecal samples collected before and 14 days after AH administration were analyzed by the McMaster method, which had an analytical sensitivity of 50 EPG [23,24,32]. Only goats with an EPG ≥200 (before AH administration) were included in the calculation. The obtained data was analyzed and interpreted using the online software eggCounts available at http://shiny.math.uzh.ch/user/furrer/shinyas/shiny-eggCounts/ (accessed on 2 April 2024) [33,34].

### 2.5. Statistical Analyses

The frequency and prevalence with its 95% confidence interval (CI) of identified internal parasites were calculated for the overall goat population and stratified by age (young 6–12 months and adults >12 months) and season (autumn and spring). Differences between age groups and seasons were evaluated by the Chi-square test with Yates correction. The level of statistical significance was set at *p*-value < 0.05. Statistical analyses were performed by using the EpiInfo 2000 program (CDC, Atlanta, GA, USA, 2000) [35].

## 3. Results

### 3.1. Occurrence of Endoparasites and GIS Species

All goats were infected with at least one internal parasite. On day 0, the most prevalent internal parasites were *Eimeria* spp. (95.5%), and GIS (91.0%), followed by *P. rufescens*/*M. capillaris* (77.6%), *Dyctiocaulus filaria* (56.7%), and *Moniezia* spp. (43.3%). On day 14, similar rates of *Eimeria* spp. (96.2%, *p* = 0.85) and GIS (81.1%, *p* = 0.11) were recorded, while rates of *P. rufescens/M. capillaris* (30.2%, *p* < 0.00001), *Dyctiocaulus filaria* (11.3%, *p* < 0.00001), and *Moniezia* spp. (13.2%, *p* = 0.0004) significantly decreased (Table 2).

The overall prevalence of endoparasitic infections was similar between age groups at both timepoints, except for *P. rufescens/M. capillaris* infection, which was significantly higher in adult goats compared to young goats after AH administration (Table 3). During the autumn season, significantly higher rates of infections with lungworms and tapeworms were recorded before AH administration as compared to spring. Table 3 includes more details.

Overall, the mean OPG/EPG values were less than 1000 per gram of fecal sample and tended to be higher in young goats compared to adults, and in autumn than in spring; however, the differences were not statistically significant (Table 4).

Before AH administration, six out of the 13 GIS species tested were identified by PCR in all herds but H3, as shown in Table 5. Fourteen days after AH administration, H1 tested positive for *H. contortus* infection, while H2 and H5 tested positive for *T. colubriformis* infection. More details on the GIS species identified in each herd are shown in Table 5.

### 3.2. Outcomes of AR Evaluation

Table 6 shows the results of the FAMACHA score, EPG before and after eprinomectin or albendazole administration, and FECRT outcomes in herds H1, H2, and H5. The obtained values for FAMACHA score before AH administration were higher than 3, indicating the need for deworming. At 14 days after AH administration, the FAMACHA score had similar values. The FECRT rates were 38.42% and 54.71% in H1 and H2, respectively, which were treated with eprinomectin, and 9.78% in H5, which was treated with albendazole. According to these values, the GIS populations identified in these herds are resistant to the administered AHs (Table 6).

## 4. Discussion

The results of the present study provide an overview of the internal parasitic profile and the major concerns that should be addressed in goats reared under extensive systems prophylactically treated with AHs without prior parasitic profile assessment and susceptibility testing. Although the study was conducted in five herds from northwestern Romania, which was among the top three goat farming countries in Europe in 2022 [36], its design provides a useful model on how to conduct anthelmintic susceptibility testing in order to develop effective parasite control programs in goat farming worldwide. Firstly, our study revealed a high prevalence (100%) of endoparasitic infection, indicating an urgent need for intervention. Furthermore, the presence of AR among GIS poses a significant challenge to effective parasitic control, emphasizing the necessity of identifying alternative management strategies.

*Eimeria* spp. was the most common endoparasite (95.5%) identified in the present study, followed by GIS (91.0%), *P. rufescens*/*M. capillaris* (77.6%), *Dyctiocaulus filaria* (56.7%), and *Moniezia* spp. (43.3%). The high rate of *Eimeria* spp. infection is consistent with recent reports from goat farms in other European countries, such as Slovakia (85%) [6], northern Italy (93%) [7], northeastern Italy (78%) [37], the Czech Republic (90%), and Poland (80%) [8,38]. Using the classification of *Eimeria*-free, low (<1800 OPG), medium (1800–6000 OPG), and high (>6000 OPG) proposed by Idris et al. (2018) [39], the intensity of *Eimeria* spp. infection was, on average, low (OPG 823 ± 202) in our study. Across and within European countries, infection intensity varies substantially, with Poland reporting a low intensity (550 OPG) [38], Slovakia medium (1816 OPG) [6], and Northern Italy ranging from low (51 OPG) to high (up to 43,004 OPG) [7,37]. The observed variations among farms are likely attributable to differences in individual management practices. Furthermore, it is important to distinguish between less pathogenic *Eimeria* spp. to better assess the infection’s impact.

The high rate of GIS infection recorded in this study is similar to data from Slovakia (90%) [6,40], the Czech Republic (93%) [8], and Poland (87%) [38], and lower than in northeastern Italy (38%) [37], possibly due to different management practices, climate, and/or type of GIS. According to Soulsby (1982) [41], who classified FEC values of GIS into GIS-free, low (<500 EPG), medium (500–1000 EPG), and high (>1000 EPG), the GIS infection intensity before AH administration was medium in both young and adult goats (mean values: 800 and 539 EPG, respectively). Various intensities have also been reported in northern Italy (34–2085 EPG) [7], northeastern Italy (484 EPG) [37], and Slovakia (1493 EPG) [6].

Regarding age-related differences in infections’ occurrence and intensity before AH administration, *Eimeria* spp. infection was observed at similar rates in both age groups (>90%), with a low infection intensity (OPG < 1800). However, young goats shed twice as many oocysts as adult goats, which is expected since animals develop resistance following repeated exposures. A similar trend was reported by Lambertz et al. (2018) in multiple goat farms in northern Italy [7]. No statistically significant differences in the rate of endoparasitic infections were observed across different age groups. However, seasonal variations in infection rates were evident. The higher infection rates with lungworms and tapeworms in autumn compared to spring can be explained by several factors, including overgrazing, which heightens parasite exposure later in the grazing season, and favorable autumn climatic conditions that prolong the activity of intermediate hosts [42]. Furthermore, routine spring deworming may temporarily reduce parasite burdens, leading to a resurgence in autumn. Similarly, the occurrence of *Eimeria* spp. infections tend to increase in autumn due to higher humidity, which promotes oocyst sporulation [43].

The prevalence of GIS infection did not significantly differ between age groups. In small ruminants, the most economically significant GIS infections are caused by *Haemonchus* spp., *Teladorsagia* spp., *Ostertagia* spp., *Trichostrongylus* spp., *Mecistocirrus* spp., *Nematodirus* spp., and *Cooperia* spp., as well as *Bunostomum* spp. and *Oesophagostomum* spp. [44,45,46]. In the present study, six of these species were identified in four of the five herds before AH administration. This composition of strongylid species is common among goat herds, as also observed in studies from Slovakia [6] and the Czech Republic [8]. *Haemonchus contortus* is particularly pathogenic due to its blood-feeding activity [17]. Although this parasite was identified in only two herds, it requires special attention as it thrives in the humid and warm climatic conditions present in some regions of our country [47]. Fourteen days after AH administration, H1 tested positive for *H. contortus* infection, while H2 and H5 tested positive for *T. colubriformis* infection. These findings may imply that *H. contortus* identified in H1 is resistant to eprinomectin, and *T. colubriformis* in H2 and H5 is resistant to eprinomectin and albendazole, respectively. Although definitive conclusions cannot be drawn since reinfections may have also occurred, these findings emphasize the importance of anthelmintic susceptibility testing to inform treatment decisions, particularly in herds that have not responded to previous AH administrations.

The presence of GIS in the goat populations studied represents a potential constraint in their health and production that could result in important economic losses. Furthermore, AR to eprinomectin and albendazole identified through FECRT complicates even more herd health and productivity management. These findings are consistent with those reported by Potârniche et al. (2021) regarding GIS resistance to benzimidazoles and macrocyclic lactones in a dairy goat herd from the same region [21], suggesting that GIS AR is becoming a serious issue in our country, contributing to persistent parasitic infections and associated health complications.

In Europe, AR to benzimidazoles and macrocyclic lactones have been recently reported in goats from Poland [11,14,15], the Czech Republic [16], Slovakia [17,18], and France [19,20]. Several reports have also confirmed MDR to these agents and to levamisole, raising further concerns. Since AHs within a specific drug class share the same mechanism of action, resistance to one AH class may lead to cross-resistance within the same class [48,49]. Cross-resistance may also develop between AHs of different classes if they have similar targets [48]. For example, cross-resistance between benzimidazoles and macrocyclic lactones is plausible due to the identical nucleotide changes in the β-tubulin isotype 1 gene detected in both ivermectin-resistant *H. contortus* and benzimidazole-resistant strains [50].

The results of this study represent only the “tip of the iceberg”. A major issue is the lack of awareness among farmers and veterinarians regarding the factors contributing to AR development and the spread of ruminant parasites. One key factor accelerating the AR is the administration of inadequate AH doses [51]. In Romania, farmers often use AHs that are not specifically approved for goats, extrapolating dosages from other species. However, goats metabolize AHs more quickly than other animals, necessitating higher doses [52]. A questionnaire-based survey found that more than 78% of Romanian goat farmers (183/234) were unaware that AH doses differ between goats and sheep. Additionally, only 2.6% (6/234) of goat farmers calculated AH dosage based on individual body weight, while most relied on visual estimation—a method prone to dosing errors [53]. Overuse and misuse of AH, including overdosing, underdosing, or frequent treatment, are key drivers for the selection of resistant GIS populations [49]. Moreover, the lack of registered AHs for goats results in repeated use of the same AH class, further increasing resistance risks [53].

To mitigate AR development in goats, farm management strategies should focus on preventing parasite infections, maintaining low infection pressure, preserving refugia, using AHs correctly in terms of dosing and administration, basing AH selection on fecal egg count rather than a fixed schedule, rotating AH classes, and implementing quarantine measures for newly acquired animals. Integrated parasite management, including rotational grazing, selective breeding for resistance, and biological control, should also be prioritized [49].

The present study has several limitations. Firstly, the sample size was limited, and the follow-up period was short, which did not allow us to assess MDR. Secondly, relying solely on FECRT to evaluate the efficacy of AHs in goats could lead to an underestimation of the AR level on farms where resistant alleles are found in only a small proportion of the GIS population [54]. However, this data provides insights into goat parasitism and AR in Romania, enhancing the existing body of knowledge in Eastern Europe. Further research is needed with longer follow-up periods to assess MDR.

## 5. Conclusions

The high rates of GIS and other internal parasitic infections (*Eimeria* spp., lungworms, *Moniezia* spp.), combined with the suboptimal efficacy of both albendazole and eprinomectin observed in this study, raise significant concerns for goat health and productivity in Romania. Inadequate control of GIS infections can result in substantial economic losses for farmers. These findings underscore the urgent need to move away from routine prophylactic treatments and emphasize the importance of evidence-based parasite control strategies. Parasite control programs should prioritize anthelmintic susceptibility testing to guide treatment decisions and ensure the use of effective drugs. Furthermore, exploring alternative strategies, such as integrated parasite management programs, is crucial in maintaining the long-term sustainability of goat farming.

## Figures and Tables

**Table 1:** Specific primers for gastrointestinal strongyle species used in PCR.

Parasite Species	Primer	5′-3′ Primer Sequence	Amplicon Size (pb)	Hibridization Temperature
*Teladorsagia circumcincta* [29]	TeciFd3	AAACTACTACAGTGTGGCTAACATA	295–297	53 °C
	ITS2GR	GCTAAATGATATGCTTAAGTTCAGC		54 °C
*Trichostrongylus axei* [29]	TraxFd2	GATGTTAATGTTGAACGACATTAATATC	186	52 °C
	ITS2GR	GCTAAATGATATGCTTAAGTTCAGC		54 °C
*Trichostrongylus colubriformis* [29]	ITS2GF	CACGAATTGCAGACGCTTAG	232	54 °C
	TrcoRv1	ACATCATACAGGAACATTAATGTCA		52 °C
*Trichostrongylus vitrines* [29]	TrviFd1	ATGTGAACGTGTTGTCACTGTTTA	150	53 °C
	ITS2GR	GCTAAATGATATGCTTAAGTTCAGC		54 °C
*Ostertagia leptospicularis* [29]	OsleFd2	CATGCAACATAACGTTAACATAATG	196	52 °C
	ITS2GR	GCTAAATGATATGCTTAAGTTCAGC		54 °C
*Oesophagostomum venulosum* [29]	ITS2GF	CACGAATTGCAGACGCTTAG	323/327/329	54 °C
	OeveRv1	CGACTACGGTTGTCTCATTTCA		54 °C
*Cooperia curticei* [29]	CocuFd3	TAATGGCATTTGTCTACATTGGTTC	252	53 °C
	ITS2GR	GCTAAATGATATGCTTAAGTTCAGC		54 °C
*Chabertia ovina* [29]	ChovFd2	CAGCGACTAAGAATGCTTTGG	115/117	54 °C
	ITS2GR	GCTAAATGATATGCTTAAGTTCAGC		54 °C
*Haemonchus placei* [30]	HpBotuF	CCAGACCCGAGACTCGCC	459	58.5 °C
	HpBotuR	CTGAAGGTAATGTCAAAATTTCT		
*Haemonchus contortus* [31]	H ConF	CATATACATGCAACGTGATGTTATGAA	260	62 °C
	H ConR	GCTCAGGTTGCATTATACAAATGATAAA		
*Cooperia oncophora* [29]	ITS2GF	CACGAATTGCAGACGCTTAG	173	54 °C
	CoonRv1	CTATAACGGGATTTGTCAAAACAGA		53 °C
*Nematodirus fillicolis* [29]	ITS2GF	CACGAATTGCAGACGCTTAG	162	54 °C
	NefiRv1	GGGATTGACTGTTACGATGTAA		50 °C
*Nematodirus spathiger* [29]	ITS2GF	CACGAATTGCAGACGCTTAG	213	54 °C
	NespRv1	CATTCAGGAGCTTTGACACTAAT		

**Table 2:** Occurrence of identified internal parasites in goats (N = 67).

Parasitic Infections	Day 0		Day 14		*p*-Value
	% (n/N)	95% CI	% (n/N)	95% CI	
*Eimeria* spp.	95.5 (64/67)	87.6–98.5	96.2 (51/53)	87.3–99.0	0.85
*Moniezia* spp.	43.3 (29/67)	32.1–55.2	13.2 (7/53)	6.6–24.8	0.0004
Gastrointestinal strongyles	91.0 (61/67)	81.8–95.8	81.1 (43/53)	68.6–89.4	0.11
*Protostronylus rufescens/* *Mullerius capillaris*	77.6 (52/67)	66.3–85.9	30.2 (16/53)	19.5–43.5	<0.00001
*Dyctiocaulus filaria*	56.7 (38/67)	44.8–67.9	11.3 (6/53)	5.3–22.6	<0.00001
Total	100 (67/67)	94.6–100	100 (53/53)	93.2–100	0.87

Legend: N—total number of sampled goats included in the study; n—number of positive goats in a specific category; CI—confidence interval.

**Table 3:** Occurrence of identified internal parasites in goats by age category and season.

Category		*Eimeria* spp. % (n/N)	*Moniezia* spp. % (n/N)	GIS % (n/N)	*P. rufescens/* *M. capillaris* % (n/N)	*D. filaria* % (n/N)
Young	Day 0	92.9 (13/14)	64.3 (9/14) ^a^	85.7 (12/14) ^a^	64.3 (9/14) ^a^	64.3 (9/14) ^a^
	Day 14	100.0 (14/14)	21.4 (3/14)	78.6 (11/14)	0.0 (0/14)	0.0 (0/14)
Adults	Day 0	96.2 (51/53) ^a^	37.7 (20/53) ^a^	92.5 (49/53) ^a^	81.1 (43/53) ^a^	54.7 (29/53) ^a^
	Day 14	94.9 (37/39)	10.3 (4/39)	82.1 (32/39)	41.0 (16/39)	15.4 (6/39)
*p*-value	Day 0	0.85	0.14	0.80	0.325	0.734
	Day 14	0.83	0.19	0.77	0.02	0.43
Autumn	Day 0	94.7 (36/38) ^a^	71.1 (27/38) ^b^	97.4 (37/38) ^a^	100.0 (38/38) ^b^	100.0 (38/38) ^b^
	Day 14	96.9 (31/32)	6.3 (2/32)	78.1 (25/32)	15.6 (5/32)	18.8 (6/32)
Spring	Day 0	96.6 (28/29) ^a^	6.9 (2/29) ^a^	82.8 (24/29) ^a^	48.3 (14/29) ^a^	0.0 (0/29) ^a^
	Day 14	95.2 (20/21)	23.8 (5/21)	90.5 (19/21)	52.4 (11/21)	0.0 (0/21)
*p*-value	Day 0	0.810	<0.00001	0.100	<0.00001	<0.00001
	Day 14	0.76	0.06	0.24	0.004	0.13

Legend: GIS—gastrointestinal strongyles; n—number of goats in a specific category. Values with different superscripts (a, b) within a row are significantly different (*p* < 0.05).

**Table 4:** OPG/EPG values (mean ± SEM) for the infection with *Eimeria* spp. and GIS overall and by age category and season before AH administration.

	*Eimeria* spp.		GIS	
	Day 0	Day 14	Day 0	Day 14
Age category				
Young	1323 ± 717	938 ± 221	800 ± 303	382 ± 137
Adults	695 ± 178	716 ± 85	539 ± 75	584 ± 101
*p*-value	0.214	0.82	0.221	0.11
Season				
Autumn	1058 ± 345	597 ± 106	565 ± 106	325 ± 109
Spring	520 ± 120	1046 ± 140	629 ± 141	613 ± 105
*p*-value	0.190	0.001	0.714	0.04
Total	823 ± 202	783 ± 89	590 ± 84	533 ± 83

Legend: OPG/EPG—oocysts/eggs per gram fecal sample; SEM—standard error of the mean; GIS—gastrointestinal strongyles; AH—anthelmintic.

**Table 5:** GIS species identified by PCR before (day 0) and after (day 14) AH administration.

	*Haemonchus placei*	*Haemonchus contortus*	*T* *eladorsagia circumcincta*	*Trichostrongylus colubriformis*	*Oesophagostomum venulosum*	*Cooperia oncophora*
H1						
Day 0	P	P	P	P	P	N
Day 14	N	P	N	N	N	N
H2						
Day 0	P	N	P	P	P	N
Day 14	N	N	N	P	N	N
H3						
Day 0	N	N	N	N	N	N
H4						
Day 0	N	P	P	P	N	P
H5						
Day 0	P	N	P	P	P	P
Day 14	N	N	N	P	N	N
Total						
Day 0	3/5	2/5	4/5	4/5	3/5	2/5
Day 14	0/3	1/3	0/3	2/3	0/3	0/3

Legend: AH, anthelmintic; GIS, gastrointestinal strongyles; H, herd; PCR, polymerase chain reaction; P—positive; N—negative.

**Table 6:** FAMACHA score, FECR, and its 90% confidence interval in treated herds for GIS.

Herd	Drug	FAMACHA (Mean ± SEM)		EPG (Mean ± SEM)		FECR % (90% CI)	Classification
		Day 0	Day 14	Day 0	Day 14		
H1 (N = 15)	EPR	3.2 ± 0.2	3.2 ± 0.2	2539 ± 595	1267 ± 195	38.42 (23.4–71.8)	Resistant
H2 (N = 71)	EPR	3.1 ± 0.0	3.1 ± 0.0	605 ± 118	355 ± 132	54.71 (−4.1–75.1)	Resistant
H5 (N = 21)	ALB	3.6 ± 0.2	3.6 ± 0.2	750 ± 192	486 ± 89	9.78 (−1.7–64.8)	Resistant

Legend: FECR—fecal egg count reduction; GIS—gastrointestinal strongyles; EPG—eggs per gram; SEM—standard error of the mean; EPR—eprinomectin; ALB—albendazole; CI—confidence interval.

## Data Availability

The original contributions presented in the study are included in the article; further inquiries can be directed to the corresponding author.
